# Such Is Fame

**Published:** 1886-04

**Authors:** 


SUCH IS FAME(?)

"What's in a name?" depends on whose name it is.

Yes, its a delicate matter, and we do not like to speak about it—out loud. But who ever saw a man who was not sensative about his name? Be it ever so ugly or ever so common, one likes to have it spelled correctly. We were once buttonholed an hour by a gentleman who insisted on pointing out (with finger emphasis) the vast difference, (not between *tweedle dum and tweedle dee*), but betwen Clanahan and Cl*e*nahan; his name,he said,was spelled with an *e*—Cl*e*n-a-han. He a-l-w-a-y-s spelled it so, and his grandfather before him. We are quite sure we will *never* forget the difference, nor the occasion; and what's more, *we* hadn't called him "Clanahan;" we hadn't called him at all; he called *us*. Now, a friend, an old acquaintance who should have known better, sent us a draft payable to "F. E. Daniels"—compelling us to sign "out of our own name;" and when in replying we stuck an s on to the end of *his* name, he got mad. Hang a fellow that can't take a joke; but it illustrates what we have just said, men are sensative about their names. During the war we heard a young fellow say it "*would be* sweet to die for one's country, if it were not for having one's name spelled wrong in the paper."

Now, all this is by way of preface. We do want *so much* to impress our readers, and especially our enchanges, whose editors see our name correctly spelled in every number of the JOURNAL, that our name is *Daniel* and *not* "Daniels." Unlike Daniel Webster's man whom he compared to a mule, saying he "had no pride of ancestry and no hope of posterity," we have both, and we insist on being addressed by our correct name—the old Virginia name--on whose 'scutcheon there has never been, so far as we are aware, a stain. We confess though, what sawed us off more than being addressed as Dr. "Daniels," (it knocked the conceit out of us considerably) was, receiving one of our exchanges last week addressed to "DAVID'S" TEXAS MEDICAL JOURNAL." It fatigued us, quite.

